# Pathologic Evaluation of Endoscopically Resected Non-Ampullary Duodenal Lesions: A Single Center Experience

**DOI:** 10.5146/tjpath.2019.01474

**Published:** 2020-05-15

**Authors:** Orhun Çığ Taşkın, Fatih Aslan, Çisel Aydın Meriçöz, Volkan Adsay, Yersu Kapran

**Affiliations:** Departments of Pathology, Koç University Hospital, Istanbul, Turkey; Departments of Gastroenterology, Koç University Hospital, Istanbul, Turkey

**Keywords:** Duodenum, Endoscopic resection, Endoscopic submucosal dissection, Endoscopic mucosal resection, Histopathology

## Abstract

***Objective:*** Endoscopic resections are increasingly being used for superficial gastrointestinal lesions. However, application of these techniques in the duodenum remains challenging, due to the technical difficulties and high complication rates. This study projects a western tertiary center’s experience in the endoscopic treatment and diagnostic workup of 19 cases of non-ampullary duodenal lesions.

***Material and Method:*** Specimens (12 endoscopic mucosal resections, 6 endoscopic submucosal dissections, and one endoscopic full-thickness resection) were processed following a strict protocol (photographed, mapped digitally and submitted totally) for histopathologic examination. Clinicopathologic characteristics, margin status and follow-up information were analyzed.

***Results:*** The mean age of the 16 patients was 52 years (range: 22-81). Mean lesion size was 1.4 cm (range: 0.3-3.6 cm) for all cases, 2 cm for endoscopic submucosal dissections and 1.1 cm for endoscopic mucosal resections. Mean number of blocks submitted was 4/case. Seven neuroendocrine tumors, 3 tubulovillous adenomas were diagnosed along with nine benign lesions. For endoscopic submucosal dissections, en-bloc and R0 resection rates were 100% (n=6/6) and 83% (n=5/6); for endoscopic mucosal resections, they were 92% (n=11/12) and 83% (n=10/12), respectively. Only one patient had procedure-related late perforation that was managed endoscopically. No mortality was encountered.

***Conclusion:*** Duodenal endoscopic resections proved successful, safe and feasible methods in a tertiary center. The pathologist’s role is to designate the accurate diagnosis, related histopathologic parameters and margin status. The gross protocol was found to be essential in evaluating specimen margins and orientation, as well as in size measurement. We recommend following a standardized approach including gross photography and digital mapping when handling these specimens, for both diagnostic and data collection purposes.

## INTRODUCTION

Duodenal polyps are encountered in up to 4.6% of patients referred for upper gastrointestinal endoscopy (1). They can present as pedunculated or sessile lesions that occur sporadically or in the setting of familial polyposis (2,3). Symptomatology, histopathology and endoscopic features have important roles in the management of duodenal polyps.

Since the introduction of endoscopic mucosal resection (EMR) and endoscopic submucosal dissection (ESD) in Japan to treat gastrointestinal neoplasms (4,5), endoscopic resections (ER) are increasingly being used as a therapeutic option for various type of malignant and benign lesions throughout the gastrointestinal tract (6–11). EMR is a relatively simpler technique that allows the removal of lesions limited to the mucosa. The main disadvantage of EMR is the higher rate of piecemeal resection, especially for larger lesions. Piecemeal resections not only increase the recurrence rates and necessitate further therapy but may also jeopardize histopathological evaluation of the target lesion. ESD, on the other hand, aims the ‘en-bloc’ removal of larger and deeper lesions, allowing more accurate histopathological staging and better chance of cure with very low recurrence rate. However, ESD is a technically challenging procedure associated with a higher perforation rate (12,13).

ERs are reported to be feasible, safe and therapeutic for duodenal lesions as well (14–16). Considering the fact that the alternative surgical option for duodenal lesions is usually Whipple procedure that is associated with significant morbidity and mortality, ERs are increasingly being encouraged for superficial duodenal lesions, especially adenomas (17). Nevertheless, application of these techniques in the duodenum remains challenging due to technical difficulties and relatively high complication rates (9,18–20).

Our study included 19 cases of non-ampullary duodenal ERs performed and histopathologically examined at our institution. We aimed to present their diagnostic spectrum along with their clinicopathologic characteristics and margin status, and then compare those with the data in the literature. Combined with the short follow-up information and complication rates, we believe our results will emphasize the feasibility of ER for different types of duodenal lesions. In addition, we followed a strict protocol (that included both the endoscopy suite and the gross room) that helped the diagnostic process when handling these specimens.

## MATERIALS and METHODS

### Case Selection, Clinicopathologic Features and Follow-Up Information

A total of 19 consecutive cases of endoscopic resections involving non-ampullary duodenum, diagnosed between 2017 and 2019, were retrieved from the digital records of the pathology department. All pathology slides of the cases were retrieved from the archive and reviewed. Clinical and pathological features were collected from the pathology reports and gastroenterology files. A search was conducted in the digital records of the institution to obtain follow-up information on each patient.

### Pathological Analysis

**Figure 1:**
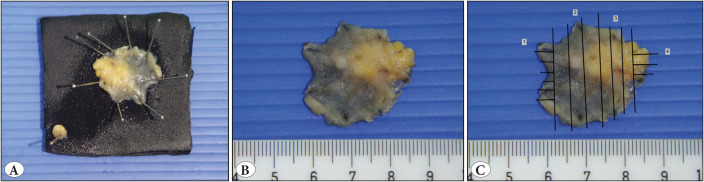
**A)** Prior to submission to the pathology department, all specimens were fixed using pins and oriented by the endoscopist in the endoscopy suite. **B)** In the gross room, specimens were photographed, **C)** grossed and mapped digitally according to the order of sampling. All specimens were totally sampled. The smaller lesion (A, buttom left) is sampled separately (not shown in the figure).

**Gross Sectioning:** Resections were performed by the same endoscopist (FA) and prepped in the endoscopy suite with pins for proper orientation. Following 12-24 hours of formalin fixation, the margins were inked; specimens were photographed and grossly inspected. Gross features (macroscopic type of the lesion, dimensions and margin status) were documented. Serial sections of 2-3 mm thickness were submitted, with a maximum of 3-4 tissues per block. Specimens were mapped on their gross digital photograph and submitted totally for histopathologic examination. Horizontal (mucosal) margins were submitted perpendicular (Figure 1). Serial and/or deeper sections were ordered when and if necessary.

**Diagnostic Process and Surgical Margins:** Specimens were diagnosed by dedicated gastrointestinal pathologists, who possessed expertise in handling and reporting endoscopic resection specimens throughout the gastrointestinal tract. Consensus decisions of at least two pathologists were made to overcome challenges in the diagnostic process and in the interpretation of histologic features. Surgical margins (vertical and horizontal) were reported as negative, when all margins were free of the lesion (R0 resection); positive when the lesion touched the inked horizontal (mucosal) and/or vertical (deep) margin (R1 resection); and equivocal, when the specimen was not fit for margin assessment, due to piecemeal excision or procedural defects (Rx resection).

### Additional Information

This study was approved by the institutional review board. Helsinki principles were respected during the study and the patients’ confidential data were kept according to their guidelines. Due to the retrospective nature of the study, written informed consent was not obtained. Pathologic data of the patients were de-identified and analyzed anonymously.

## RESULTS

A total number of 19 cases from 16 patients were retrieved. Among these 19 endoscopic resections, 12 were EMRs, 6 were ESDs, and one was endoscopic full-thickness resection. All specimens were submitted in total, with a mean number of blocks of 4 per case (range: 1-13).

### Clinical Information, Localization, Size

The mean age of the 16 patients (9 males, 7 females) was 52 years (range: 22-81). One patient was under clinical surveillance for Peutz-Jeghers syndrome.

All 19 cases were localized in the non-ampullary duodenum (6 in the bulbus, 12 in the second part, one in the third part). Mean lesion size for all cases was 1.4 cm (range: 0.3-3.6 cm). Mean lesion sizes for ESDs and EMRs were 2 cm and 1.1 cm, respectively. One EMR case was piecemeal excision, where the lesion size could not be measured (but was reported as 5 cm endoscopically).

### Diagnostic Spectrum

**Figure 2:**
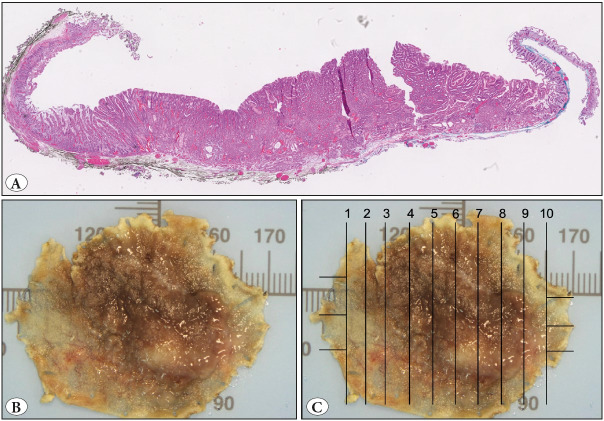
**A)** An ectopic gastric tissue, mostly composed of foveolar and oxyntic mucosa (H&E; x1) **B)** forming a flat/granular lesion of 3.3 cm in the third part of duodenum. **C)** This specimen was submitted totally in 10 blocks.

**Figure 3:**
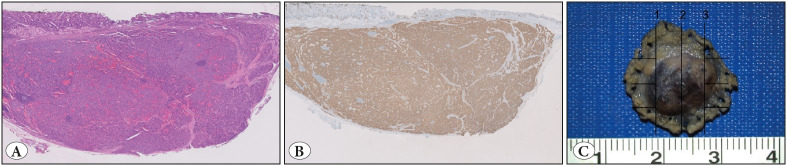
**A)** A well-demarcated submucosal nodule (H&E; x1), **B)** showing diffuse positivity for chromogranin (IHC; x1), consistent with a neuroendocrine tumor. **C)** Notice the pin marks around the nodule in the gross photograph that is taken from the vertical margin surface.

**Figure 4:**
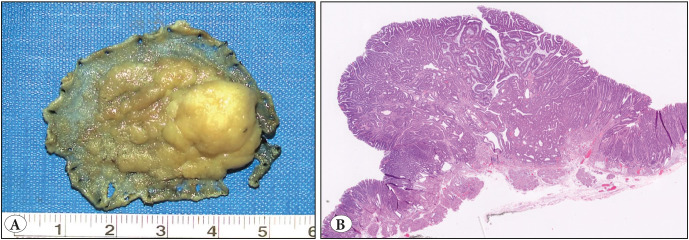
**A)** A 3.6 cm nodule, excised en-bloc. **B)** Histologic features were consistent with tubulovillous adenoma (H&E; x1).

**Figure 5:**
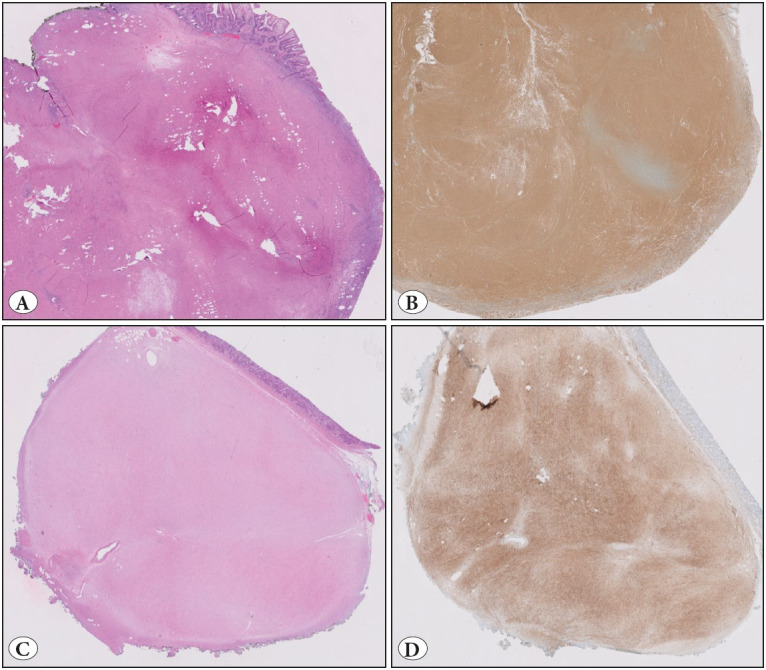
**A,C)** Two submucosal nodules, showing histologic features of leiomyoma and inflammatory fibroid polyp, respectively (H&E; x1). **B)** Diffuse positivity for smooth muscle actin (IHC; x1) and **D)** CD34 (IHC; x1) is consistent with the diagnosis.

There were 7 well differentiated neuroendocrine tumors (Grade 1-2), 3 tubulovillous adenomas (one including an intramucosal adenocarcinoma), 2 hamartomatous polyps (same patient), 2 pancreatic and 1 gastric heterotopia, 1 inflammatory fibroid polyp, 1 lipoma, 1 leiomyoma and 1 polypoid duodenitis diagnosed (Figure 2 Figure 3 Figure 4 Figure 5).

### En-Bloc Resection and Margin Status

18 of 19 cases were en-bloc resections and only one EMR case was piecemeal [en-bloc resection rates were 100% (n=6/6) and 92% (n=11/12) for ESD and EMR, respectively]. 16 of the 19 cases were R0 resections (R0 resection rates were 83% (n=5/6) and 83% (n=10/12) for ESD and EMR, respectively).

### Follow-Up Information

Late perforation (first week) related to the procedure has occurred in the one piecemeal EMR case (diagnosed as tubulovillous adenoma with intramucosal adenocarcinoma, measured 5 cm endoscopically) and managed endoscopically. That same patient and 4 others had follow-up endoscopies that were negative for a residual lesion. No mortality was encountered. Clinicopathological features of all cases are summarized in Table 1.

**Table 1:** Clinicopathologic features of duodenal endoscopic resection specimens.

**Case no.**	**ER type**	**Age**	**Sex**	**Localization**	**Diagnosis**	**Lesion size (cm)**	**LVI**	**Margin Status**	**Blocks submitted**
1	EFTR	75	F	Bulbus	Inflammatory fibroid polyp	1.4		R0	7
2	EMR	52	M	Second part	Neuroendocrine tumor (Grade 1)	0.5	No	R0	1
3	ESD	39	M	Second part	Tubulovillous adenoma (with high grade dysplasia)	3.6		R0	13
4	ESD	67	M	Bulbus	Neuroendocrine tumor (Grade 1)	1	No	R0	4
5	ESD	63	F	Bulbus	Neuroendocrine tumor (Grade 2)	0.3	No	R1 (VM+)	1
6	ESD	41	M	Third part	Ectopic stomach	3.3		R0	10
7	ESD	52	M	Second part	Ectopic pancreas	1.8		R0	4
8	ESD	32	F	Second part	Tubulovillous adenoma	2.2		R0	4
9	EMR	55	M	Bulbus	Neuroendocrine tumor (Grade 1)	0.4	No	R0	1
10	EMR	54	M	Second part	Leiomyoma	3		R0	6
11	EMR	37	F	Second part	Ectopic stomach and pancreas	1.1		R0	1
12	EMR	81	M	Second part	Intramucosal adenocarcinoma and tubulovillous adenoma	Piecemeal	No	Rx (Piecemeal)	8
13	EMR	63	M	Second part	Lipoma	1		R0	1
14	EMR	40	F	Second part	Polypoid duodenitis	1.1		R0	1
15	EMR	53	F	Second part	Neuroendocrine tumor (Grade 2)	0.7	No	R0	1
16	EMR	55	F	Bulbus	Neuroendocrine tumor (Grade 1)	0.3	No	R1 (HM+)	1
17	EMR	55	F	Bulbus	Neuroendocrine tumor (Grade 1)	0.3	No	R0	3
18	EMR	22	M	Second part	Hamartomatous polyp	1.5		R0	1
19	EMR	22	M	Second part	Hamartomatous polyp	1.7		R0	4

**ER:** Endoscopic resection, **LVI:** Lymphovascular invasion, **EFTR:** Endoscopic full-thickness resection, **ESD:** Endoscopic submucosal dissection, **EMR:** Endoscopic mucosal resection, **VM:** Vertical margin, **HM:** Horizontal margin.

## DISCUSSION

Duodenal polyps are rare lesions that may need removal, depending on their clinical, pathological and endoscopic features. This study projects, mainly from the pathology perspective, a western tertiary referral center’s two years’ experience in the endoscopic treatment and diagnostic workup of non-ampullary duodenal lesions.

The majority of the literature on advanced duodenal ERs originates from the Far East. For example, Hoteya et al. studied a large series of 129 non-ampullary duodenal ERs (74 ESD vs. 55 EMR), and concluded that duodenal ESD is useful for larger (>20 mm) lesions (21). As for the West, the contribution to the literature is relatively recent: only a handful of duodenal ERs were reported recently (22–24), yet series with comparable sizes (i.e. n=166 duodenal ERs in Pérez-Cuadrado-Robles et al’s study) are being published from specialized referral centers (25,26). The main reason for this is that, similar to other sites of the gastrointestinal tract, the western experience with duodenal ESD and EMR was limited compared to the Far East (22–24,27–32), due to flat learning curve of these techniques and lack of training facilities for the endoscopists in the west (17,33–35). In fact, as reported by Yamamoto et al., even in Japan where these procedures were developed and mastered, duodenal ESDs are performed in only a few institutions, due to the high risk of complications (19). Having mentioned these, the main limitation of this study was the relatively small size of the cohort.

ERs are reported to be useful in the treatment of various benign and malignant duodenal lesions, including intramucosal adenocarcinomas, adenomas, lipomas and neuroendocrine tumors (20,36–39). The required specific technique (e.g. ESD vs. EMR) is selected mostly according to the size and the location of the lesion (21). Nevertheless, a management strategy following the endoscopic removal of pre-malignant or malignant lesions has not yet been clearly identified, due to lack of scientific evidence. The European Society of Gastrointestinal Endoscopy suggests, with low quality of evidence, consideration of surgical therapy, when carcinoma is spotted in the specimen, particularly in the submucosa (17). We have one case with intramucosal adenocarcinoma without submucosal invasion that is under follow-up for two years without any proof of residual or systemic malignancy (also mentioned below).

A successful endoscopic resection aims en-bloc removal of the targeted lesion, with microscopically negative surgical margins (both horizontal and vertical). This verification implies total submission of the specimen in the gross room, which, in our experience, required an average of 4 submitted blocks per case. Our cohort’s en-bloc and R0 resection rates were acceptable (100% en-bloc and 83% R0 rate for ESD; 92% en-bloc and 83% R0 rate for EMR), compared to the data in the literature (14,16,20,25,30,40).

Delayed perforation is a morbid complication of ERs, and is known to be associated with the location of the lesion (distal to ampulla of Vater) and resection method (piecemeal EMR or ESD) in the duodenum (41). Accordingly, in our study group, a delayed perforation occurred in a piecemeal EMR patient, with the diagnosis of adenoma with intramucosal adenocarcinoma. Fortunately, the patient was successfully managed endoscopically, and no mortality has encountered. In addition, follow-up endoscopic examinations, also verified by histology, did not show any residual lesions.

The gross photos and mapping were crucial in evaluating the margins and orientation of the specimen, as well as in size measurement. The established protocol followed both by the endoscopy suite and pathology gross room (including proper orientation and fixation, taking/mapping gross photographs, submitting in total) allowed comfortable and accurate evaluation of the findings.

In conclusion, duodenal ESD and EMR proved to be successful, safe and feasible methods in a tertiary center with enough expertise in the technique and in the management of the possible complications. The pathologist’s role is to designate the accurate diagnosis of the lesion, along with the margin status and histopathologic parameters specific to the diagnosed entity. Due to the rareness of these specimens, we highly recommend following a systematic and standardized approach in handling and reporting cases of duodenal ERs, using gross photography/mapping, for both diagnostic and data collection purposes.

## Conflict of Interest

The authors declare no confict of interest.
